# Association of mitral annulus calcification, aortic valve calcification with carotid intima media thickness

**DOI:** 10.1186/1476-7120-2-19

**Published:** 2004-10-08

**Authors:** Luca Sgorbini, Angelo Scuteri, Massimo Leggio, Francesco Leggio

**Affiliations:** 1 Cardiologic Unit I.N.R.C.A.-I.R.C.C.S. Via Cassia 1167, 00100 ROMA, ITALY; 2 Geriatric Unit I.N.R.C.A.-I.R.C.C.S. Via Cassia 1167, 00100 ROMA, ITALY

**Keywords:** carotid artery disease, heart disease, atherosclerosis, imaging

## Abstract

**Background:**

Mitral annular calcification (MAC) and aortic annular calcification (AVC) may represent a manifestation of generalized atherosclerosis in the elederly. Alterations in vascular structure, as indexed by the intima media thickness (IMT), are also recognized as independent predictors of adverse cardiovascular outcomes.

**Aim:**

To examine the relationship between the degree of calcification at mitral and/or aortic valve annulus and large artery structure (thickness).

**Methods:**

We evaluated 102 consecutive patients who underwent transthoracic echocardiography and carotid artery echoDoppler for various indications; variables measured were: systemic blood pressure (BP), pulse pressure (PP=SBP-DBP), body mass index (BMI), fasting glucose, total, HDL, LDL chlolesterol, triglycerides, cIMT. The patients were divided according to a grading of valvular/annular lesions independent scores based on acoustic densitometry: 1 = annular/valvular sclerosis/calcification absence; 2 = annular/valvular sclerosis; 3 = annular calcification; 4 = annular-valvular calcification; 5 = valvular calcification with no recognition of the leaflets.

**Results:**

Patient score was the highest observed for either valvular/annulus. Mean cIMT increased linearly with increasing valvular calcification score, ranging from 3.9 ± 0.48 mm in controls to 12.9 ± 1.8 mm in those subjects scored 5 (p < 0.0001). In the first to fourth quartile of cIMT values the respective maximal percentual of score were: score 1: 76.1%, score 2: 70.1%, score 4: 54.3% and score 5: 69.5% (p > 0.0001).

**Conclusion:**

MAC and AVC score can identify subgroups of patients with different cIMT values which indicate different incidence and prevalence of systemic artery diseases. This data may confirm MAC-AVC as a useful important diagnostic parameter of systemic atherosclerotic disease.

## Background

Mitral annular calcification (MAC) and aortic annular calcification (AVC) are observed in populations that develop significant atherosclerosis and more frequently in the elderly [1]. Previous pathological studies have suggested they represent a degenerative process that progresses with advancing age [2-4]. Consistently with this hypothesis, several ultrasound cardiovascular studies demonstrated a significant association between MAC and coronary artery disease, aortic atheroma and peripheral arterial atherosclerotic disease [5-8].

Currently, there are no accurate and standardized methods to quantify the degree of MAC an AVC; many studies have been performed with the aim to detect categorical scoring systems derived from echocardiographic annular-valvular morphology [9-12].

Alterations in vascular structure such as increased arterial wall thickness, as indexed by the intima media thickness (IMT), are also increasingly recognized as significant independent predictors of adverse cardiovascular outcomes [13-17].

We therefore undertook a cross-sectional study to examine the relationship between the degree of calcification at mitral and/or aortic valve annulus and large artery structure (thickness).

## Subjects and Methods

We evaluated 128 consecutive patients who underwent transthoracic echocardiography and carotid artery echoDoppler for various indications. Patients with significant common carotid artery stenosis, rheumatic valvular disease, cardiomyopathy, prosthetic valves, ischemic heart disease and carotid artery surgery were excluded. Thus, 102 subjects were enrolled for the present study. All participating patients gave informed consent; the study protocol was approved by the institutional ethics committee.

### Variables Measured

#### Blood pressure

Blood pressure determinations were performed with subjects in the supine position, and following a ten minute quiet resting period. Blood pressure was measured in the nondominant arm with a mercury sphygmomanometer using an appropriately sized cuff. The blood pressure values used in this study are the average of the second and third measurements. Values for systolic blood pressure (SBP) and diastolic blood pressure (DBP) were defined by Korotkoff phase I and V, respectively. Hypertension was defined as either systolic or diastolic elevation of blood pressure (>140/90 mmHg) or ongoing antihypertensive pharmacological therapy. Pulse pressure was computed as PP = (SBP-DBP); mean BP was computed as MBP = DBP +(PP/3).

#### Anthropometry and smoking status

Height and weight were determined for all participants. Body mass index (BMI) was determined as body weight (kg) / height (m)^2^. Smoking status was ascertained by a questionnaire that classified each subject as a non smoker, former, or current smoker. For the purpose of the present study, current smoker status was used.

#### Plasma lipids and fasting blood glucose

Blood samples were drawn from the antecubital vein between 7 and 8 AM after an overnight fast. Subjects were not allowed to smoke, engage in significant physical activity or take medications prior to the collection of the sample. The concentrations of plasma triglycerides and total cholesterol were determined by an enzymatic method [18,19]. HDL-cholesterol levels were obtained by selective precipitation with dextran-MgCl_2 _[20]. Serum LDL-cholesterol concentrations were estimated by the Friedewald's formula [21]. Fasting plasma glucose concentration was measured. Diabetes was defined by a fasting glucose >120 mg/dl, ore use of insulin ore ipoglicemic medication.

#### Echocardiographic measurements

**Figure 1:**
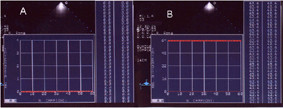
Panel A Acoustic quantification-densitometry (AD): echogray scale, black = 0 dB; Panel B echogray scale, white = 64 dB.

**Figure 2:**
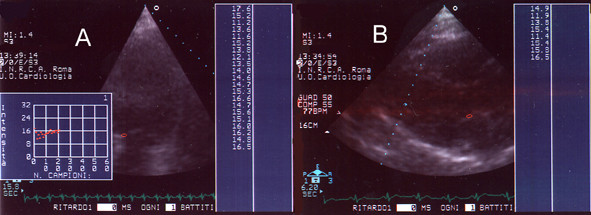
**Score 1. **Panel A: AD of mitral annulus (apical scan); Panel B: AD of aortic valve (parasternal scan).

**Figure 3:**
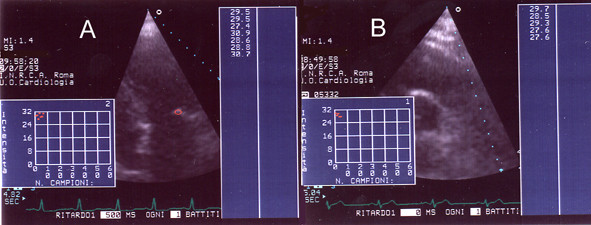
**Score 2. **Panel A: AD of mitral valve (apical scan); Panel B: AD of aortic valve (parasternal scan: short axis).

**Figure 4:**
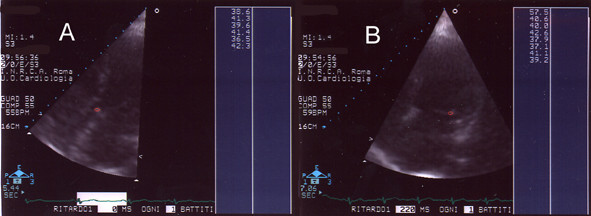
**Score 3. **Panel A: AD of aortic valve (apical scan); Panel B: AD of mitral valve (apical scan).

**Figure 5:**
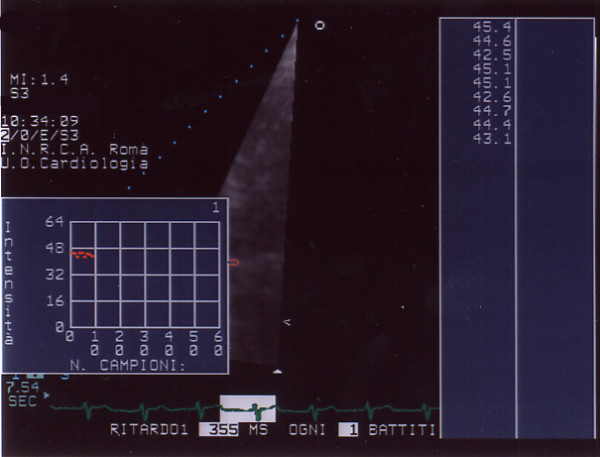
**Score 4. **AD of aortic valve (apical scan).

**Figure 6:**
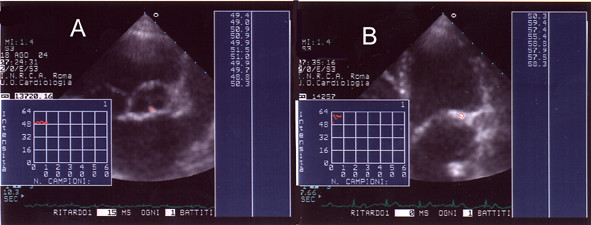
**Score 5. **Panel A: AD of aortic valve (parasternal scan: short axis); Panel B: AD of mitral valve (apical scan).

All the echocardiographic examinations were performed using the PHILIPS^® ^SONOS 5500 with a S3 probe. All patients had an adequate 2D echocardiogram. Evaluation of mitroaortic sclerosis/calcification was made, off line, with the acoustic quantification-densitometry package (PHILIPS^® ^Medical System) wich restitute values based on echogray scale (0 db = black, 64 db = white, fig. 1). From parasternal short/long axis and apical 4 – 5 chamber scans, 3 or more subsequent ECG triggered cardiac cycles were acquired (gain setting: 50, compression: 55, mechanical index: 1.4); focus and region of analysis (ROI) were positioned at the level of mitral/aortic annular/valvular sclerosis/calcification; the dimensions of ROI were 11 × 11 mm. The mitral/aortic lesions were graded by five qualitative independent scores based on 2D morphology and on acoustic densitometry values: 1= annular/valvular sclerosis/calcification absence; 10–25 dB (fig. 2); 2 = annular/valvular sclerosis; 26–35 dB (fig 3); 3 = annular calcification; 36–40 dB (fig. 4); 4 = annular-valvular calcification; 41–45 dB (fig. 5); 5 = valvular calcification with no recognition of the leaflets; > 46 dB (fig. 6). The resulting patient score was the highest observed for either valvular annulus. All the images were stored in digital format for off-line analysis and independently evaluated by three blinded operators which resulted always concordant in assigning the patient's scores.

#### Carotid Ultrasonography

High-resolution B-mode carotid ultrasonography was performed with a linear-array 5- to 10-MHz transducer. The subject lay in the supine position in a dark, quiet room. The right common carotid artery (CCA) was examined with the head tilted slightly upward in the midline position. The transducer was manipulated so that the near and far walls of the CCA were parallel to the transducer footprint and the lumen diameter was maximized in the longitudinal plane. A region 1.5 cm proximal to the carotid bifurcation was identified, and the carotid intima media thickness (cIMT) of the far wall was evaluated as the distance between the luminal-intimal interface and the medial-adventitial interface. cIMT was measured on the frozen frame of a suitable longitudinal image with the image magnified to achieve a higher resolution of detail. The cIMT measurement was obtained from 5 contiguous sites at 1-mm intervals, and the average of the 5 measurements was used for analyses. All the measurements were performed by a single sonographer.

## Statistical analysis

All analyses were performed using the SPSS 8.0 package. Data are presented as mean ± SD unless otherwise specified.

Comparison of groups based on different calcification score was made by ANOVA, followed by Bonferroni's test for all two-way comparisons, or by chi-square analysis – as appropriate. Geometric mean values of vascular end points, adjusting for traditional cardiovascular risk factors, were calculated across categorized features by means of General Linear Model.

## Results

Of the 102 patients evaluated, 24 were scored 1, 19 were score 2, 20 were score 3, 18 were score 4 and 21 were score 5. There were no statistically significant intergroup differences in age, sex distribution, total cholesterol, HDL and LDL cholesterol, smoking habits, diabetes mellitus, and positive family history of coronary artery disease (tab.1). Similarly, clinical indications for ultrasound examinations were not significantly different in the 5 score groups (tab. 2).

**Table 2:** Clinical Indications

	Score 1	Score 2	Score 3	Score 4	Score 5	All pts
Carotid murmur %	38.2	37.1	39.1	39.9	39.3	38.7
Cardiac murmur %	34.8	34.3	33.8	34.1	35	34.4
Stroke %	15.3	15.6	14.8	15.1	15.4	15.2
Cardiac surgery %	11.7	13	13.3	10.9	10.3	11.8

Systolic blood pressure showed a non statistical increase from group 1 to 5 while pulse pressure values raised significantly (tab. 1; p < 0.04).

**Table 1:** Baseline characteristics

	Score 1	Score 2	Score 3	Score 4	Score 5	All pts	ANOVA p
N° of pts	24	19	20	18	21	102	
Age yrs	68.4 ± 11.6	65.9 ± 9.8	68.4 ± 4.7	69.3 ± 4.8	65.3 ± 6.8	67.4 ± 7.5	.25
BMI Kg/m^2^	27.8 ± 5.1	29.7 ± 5.9	29.5 ± 7.8	28.4 ± 4.8	29.6 ± 4.9	29 ± 5.6	.76
Male Sex %	33	30	38	37	48	37	.31
Current Smoker %	33	21	17	32	35	27	.21
CAD Family history %	36	36	23	48	43	37	.42
Diabetes %	10	11	15	19	17	14	.18
SBP mmHg	142.5 ± 12.9	144.6 ± 11.4	140.5 ± 17.5	142.5 ± 16.3	149.1 ± 18.0	143.8 ± 15.2	.07
DBP mmHg	83.8 ± 8.2	84.6 ± 9.7	83.1 ± 10	82.8 ± 9.8	83.1 ± 9.3	83.4 ± 9.4	.29
PP mmHg	58.7 ± 10.4	60 ± 13.8	57.4 ± 11.5	59.7 ± 13.9	66 ± 17.8	60.3 ± 13.5	.03
Total Chol mg/dl	214.6 ± 42.4	204.9 ± 32.7	216.3 ± 40.6	218.3 ± 36.1	209.9 ± 36.4	212.4 ± 37.6	.62
HDL Chol mg/dl	48.4 ± 9.4	48.2 ± 10.7	52.2 ± 13.5	48.7 ± 11.3	46.7 ± 12.0	48.8 ± 11.3	.47
LDL Chol mg/dl	136.4 ± 33.7	126.9 ± 32.6	139.0 ± 37.8	140.1 ± 38.2	130.3 ± 35.9	134.5 ± 35.6	.52
TGC mg/dl	148.6 ± 56.8	149.3 ± 85.5	125.3 ± 49.1	147.3 ± 35.8	164.8 ± 49.7	147.6 ± 55.3	.19
Fasting blood Glucose mg/dl	98.9 ± 29.7	117.1 ± 45.4	113.1 ± 40.6	103.1 ± 21.9	98.0 ± 20.5	106 ± 31.6	.11
Fibrinogen mg/dl	302.1 ± 59.4	302.1 ± 56.4	325.5 ± 103.8	305.7 ± 57.4	323.0 ± 88.9	311.8 ± 73.1	.61

Pts: patients, BMI: body mass index, CAD: coronary artery disease, SBB: systolic blood pressure, DBP: diastolic blood pressure, PP: pulse pressure, Total Chol: total cholesterol, TGC: triglycerides.

**Figure 7:**
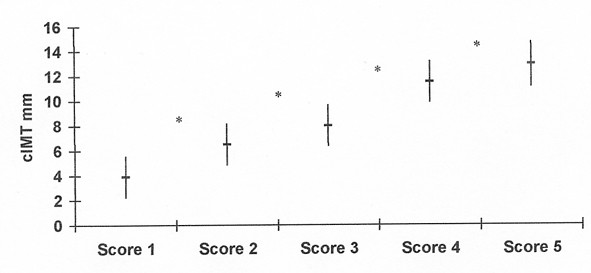
Positive association between cIMT and score groups; *p < 0.0001

Vascular characteristic of the five score groups are shown in Fig 7; mean cIMT increased linearly with increasing valvular calcification score, ranging from 3.9 ± 0.48 mm in controls to 12.9 ± 1.8 mm in those subjects with score 5 (p < 0.0001).

**Figure 8:**
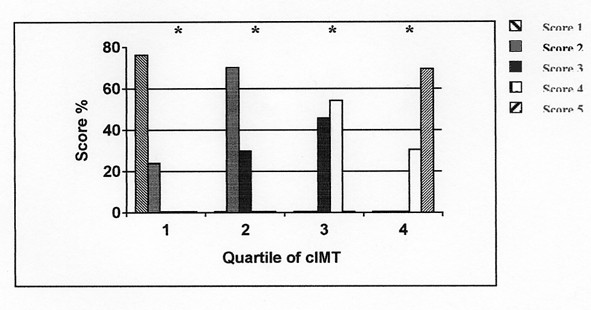
Distribution of quartiles of cIMT across scores of valvular calcification; *chi-square = p < .001

ANCOVA analysis confirmed that the association of valvular calcification score with cIMT was independent of age, sex, BMI, HDL and LDL cholesterol, smoker and diabetes (table 3). In the first to fourth quartile of cIMT values the respective maximal percentual of score were: score 1: 76.1%, score 2: 70.1%, score 4: 54.3% and score 5: 69.5% (p > 0.0001) (fig. 8); multivariate analysis showed a significant influence of systolic blood pressure from first to fourth quartile and of HDL cholesterol.

**Table 3:** ANCOVA analysis

	Age	Sex	BMI	C.S	HP	D	HDLC	LDLC
p (cIMT)	.609	.23	.479	.948	.699	.471	.625	.387

Age: years, BMI: body mass index Kg/m^2^, C.S: current smoker, HP: hypertension, D: diabetes, HDLC: HDL cholesterol mg/dl, LDLC: LDL cholesterol mg/dl.

## Discussion

The present study is the first to show a strong and significant association between the presence of MAC-AVC and cIMT values. Patients with severe MAC-AVC (scored 5) had higher values of cIMT.

Previous pathologic studies demonstrated that foam cells which represent early atherosclerotic lesions can be found in subjects already during adolescence on the endothelium of the epicardial coronary arteries, the ventricular surface of the posterior mitral leaflet and the aortic aspects of each aortic leaflet [1,22]. Experimentally-induced systemic vascular atherosclerosis is also associated with the deposition of fatty plaques on the aortic surface of aortic valve cups and the ventricular surface of the posterior mitral leaflet [22]. These findings suggest that coronary atherosclerosis, MAC and AVC have a similar aetiology and pathophisiology, particularly in the elderly: as the fatty plaques grow, their nutritional needs fail to be fulfilled and they degenerate into calcific deposits[1].

Many recent studies showed a clear association between mitral annulus calcification and the presence of aortic atheromas, atheroma thickness and carotid artery disease [4,5] these studies also found that MAC patients have higher prevalence of carotid artery stenosis [6], coronary artery stenosis [13] and peripheral artery stenosis [8], supporting the theory that MAC is a form of polisegmental atherosclerosis. Adler [23] in a recent prospective trans oesophageal echocardiographic study showed a significant association between the presence and severity of MAC and aortic atheroma, suggesting MAC as an important marker of aortic atherosclerosis; the author concluded that this association may explain in part the high prevalence of systemic emboli and stroke in patients with MAC. Even the presence and extent of AVC has been demonstrated, in many recent studies, to be directly correlated with atherosclerotic risk factors and atherosclerotic disease suggesting how AVC could represent a marker of polisegmental atherosclerosis [2,3,7,24-26].

In our study we investigated the possible association between AVC, MAC and cIMT in elderly patients; we found a clear and strong significant linear correlation between cIMT and AVC/MAC values; there were no significant influence of the considered variables on this correlation. In addition, we found a strong association of incremental values of score with first to fourth quartile of cIMT Another exclusive characteristic of our study is the creation of a semi quantitative way of AVC-MAC evaluation; in fact, scoring the evolution of AVC and MAC, we were able to correlate this parameter with the continuous variable cIMT.

Furthermore, a recent study [26] showed a strong association of aortic valve sclerosis and systemic endothelial dysfunction evaluated by ultrasonography of the brachial artery; since it is well established and demonstrated that IMT is an early marker of endothelial-organ damage and an initial precursor of systemic atherosclerotic disease [1-8] our results are consistent with those obtained by Poggianti and her collegues.

Our data indicate that AVC-MAC could be considered a form of polisegmental atherosclerosis; the semi quantitative evaluation of AVC-MAC is strongly associated cIMT; this semi quantitative way of grading mitral-aortic valvular-annular sclerosis and calcification was also able to identify the quartile of cIMT. These results indicate the score evaluations as an important echocardiographic tool for atherosclerotic disease evaluation.

### Limitations of the study

This study evaluated only elderly patients, therefore the eventual correlation of MAC-AVC and cIMT can be only supposed in younger patients; future studies are needed to demonstrate this hypothesis. We viewed cIMT as a marker for sub clinical atherosclerosis. Although the significance of carotid thickening, particularly in the distal common carotid artery, continues to be debated, its association with prevalent and future cardiovascular events has been supported by a number of studies [15-17,24,25]. Nonetheless, it should be recognized that atherosclerosis may progress in other vascular districts at different rates. Further work is need to test if these results may apply to atherosclerosis in other major vascular territories (peripheric big arteries, thoracic and abdominal aorta).

Additionally, whether our findings are sufficient to indicate a widespread use of carotid ultrasound in those with presence of MAC-AVC requires further studies.

## Conclusions

MAC-AVC presence and their scoring can be detected by transthoracic echocardiography, a simple noninvasive imaging method. Using MAC and AVC values we can identify subgroups of patients with different cIMT values, a well-established precursor of systemic atherosclerosis, which indicate different incidence and prevalence of carotid, coronary and aortic artery diseases. Therefore, mitral or aortic valve calcification should not be regarded as a natural correlate of aging, rather as markers of generalized atherosclerosis.

## List of abbreviations

MAC: Mitral annular calcification

AVC aortic annular calcification

IMT intima media thickness

cIM carotid intima media thickness

CCA right common carotid artery
